# A bridge of care: translating a Ugandan nephrology fellowship into sustainable practice in a low-resource setting

**DOI:** 10.1097/MS9.0000000000005755

**Published:** 2026-10-09

**Authors:** Louis Nyende, William O. Atubo, Kabategeki Immaculate, Donethian Mbambu, Edrine Biira, Ibrahim Bwaga

**Affiliations:** a Department of Medicine, Hoima Regional Referral Hospital, Hoima, Uganda; b Department of Internal Medicine, Kampala International University – Western Campus, Hoima, Uganda; c Department of Nephrology, Former Clinical Fellow in Adult Nephrology, McMaster University, Hamilton, Ontario, Canada

**Keywords:** capacity building, dialysis, global health, low-resource setting, nephrology fellowship, Uganda

## Abstract

**Background:**

In low- and middle-income nations, where access to renal replacement therapy is still restricted and unequal, the prevalence of chronic kidney disease (CKD) is rising quickly. To increase access to care, regional hospitals must expand their nephrology services.

**Objective:**

Using the concept of cyclical capacity building, explain how foreign fellowship training translates into local service development in a low-resource environment.

**Methods:**

The growth of nephrology services at Hoima Regional Referral Hospital in Uganda between February 2025 and February 2026, following completion of fellowship training in adult nephrology, is described in this narrative reflection and descriptive quantitative report.

**Results:**

The number of dialysis machines increased from zero to five; 2340 hemodialysis sessions were provided; sixteen chronic dialysis patients were maintained; seven cases of acute kidney injury were handled locally; two patients were referred for kidney transplants; and staff and students received structured training. These results demonstrate how circular capacity building may be applied to improve local nephrology services by converting foreign training into long-term clinical practice.

**Conclusion:**

When international fellowship training is aligned with institutional preparedness, available infrastructure, and national health policy, it can improve health systems. In low-resource environments, circular capacity building might offer a workable paradigm for long-term global health education.

## Introduction

In low- and middle-income countries (LMICs), where access to renal replacement therapy is still severely restricted, the burden of chronic kidney disease (CKD) is increasing disproportionately^[^1^]^. There are just 16 qualified nephrologists in Uganda, a country of about 45 million people, or one nephrologist for every 2.8 million people^[^2^]^. The prevalence of CKD is increasing worldwide, particularly in LMICs with limited access to renal replacement therapy^[^3–5^]^.

There was only one public-sector dialysis facility in Uganda prior to 2023: Kiruddu National Referral Hospital in Kampala. Extreme geographic misdistribution – with the bulk of experts concentrated in Kampala, the nation’s capital, leaving large areas of the country without access to renal care–exacerbates this labor shortfall. Dialysis patients from upcountry areas have little choice but to move to Kampala, pay exorbitant transportation charges for twice-weekly travel (often a 4-hour trip each way), or stop receiving treatment entirely. Although private dialysis clinics exist, most Ugandans cannot afford them, and they are primarily located in urban areas.

The Ugandan Ministry of Health initiated a dialysis expansion program in 2023 in response to this situation, working with Kiruddu National Referral Hospital. For the initial scale-up, four regional hospitals – Lira, Mbale, Mbarara, and Hoima – were chosen. Over 3 million people in the districts of Hoima, Masindi, Kikuube, Kakumiro, and Kibaale are served by Hoima Regional Referral Hospital, which is situated about 200 kilometers west of Kampala. Historically, Kampala has been the hub for dialysis services^[^6^]^.

Importantly, Hoima is a teaching hospital affiliated with Kampala International University, which trains nursing and medical students at the diploma and degree levels, providing an opportunity to incorporate renal education into the broader development of the health workforce. As one of the nation’s 14 nephrologists and a Ugandan-trained physician, I completed a clinical fellowship in adult nephrology at McMaster University in Hamilton, Ontario, Canada, from 2024 to 2025 within this framework of institutional preparedness and national policy impetus. This paper provides a thoughtful assessment of that fellowship experience and, more significantly, a thorough explanation of how, within a year of returning home, the perspectives and skills acquired were converted into quantifiable changes in the health system at Hoima Regional Referral Hospital.

The main idea of this activity is “circular capacity-building,” in which the fellow acts as both a recipient and a future transmitter of information that is tailored to local situations.

## Fellowship experience: learning in a high-resource setting

The reflection focused on methods that could be adapted for Uganda. The nephrology program at McMaster University is well known for its emphasis on interdisciplinary collaboration, competency-based teaching, and flattened educational hierarchy. I worked as a clinical fellow in a setting that prioritized patient-centered care, evidence-based medicine, and procedural excellence^[^7^]^.

### Procedural and clinical education

All areas of adult nephrology were thoroughly covered during the fellowship:

- **Acute kidney injury (AKI**): Continuous renal replacement therapy and sustained low-efficiency dialysis are two methods of managing AKI in critical care settings.
- **Chronic kidney problems**: Long-term treatment of CKD includes reducing cardiovascular risk, managing anemia, and treating mineral and bone disorders.
- **Dialysis**: prescribing hemodialysis, evaluating and managing vascular access, initiating peritoneal dialysis, and troubleshooting.
- **Kidney Transplantation**: Assessment of Deceased and Living Donors, Immunosuppression Following Transplantation, and Handling of Complications.
- **Procedures**: Peritoneal dialysis catheter placement; temporary and tunneled dialysis catheter insertion; and ultrasound-guided kidney biopsy.

### Multidisciplinary team-based healthcare

Immersion in a team-based care model is perhaps more beneficial than any single clinical skill. Nephrologists, dialysis nurses, transplant coordinators, pharmacists, nutritionists, and social workers participated in weekly interdisciplinary rounds that helped to provide complete patient care^[^8^]^. Although this methodology required many resources, it provided a paradigm for how integrated care could enhance outcomes in settings with fewer resources^[^9^]^. At McMaster, trainees are expected to actively participate in quality improvement, teaching, and clinical decision-making. This environment encourages self-assurance and equips fellows to take on leadership roles when they return home. Fellows who have received training abroad return home with the skills they acquired in the best settings, and it takes time for them to adjust to their home country. To put what they’ve learned into practice, they must be patient and detail-oriented^[^10^]^.

### Conscious filtering: finding lessons that can be applied

I maintained a deliberate practice of determining which aspects of Canadian nephrology care could be practically applied in Uganda during the fellowship. This method of “conscious filtering” took into account the following:

- Which abilities best handle the most demanding circumstances in my environment?
- Which initiatives are practical given the resources and infrastructure at hand?
- Which care models can be modified with the least amount of extra funding?
- How can I train others so that my training has a greater impact?

In order to bridge the gap between the two radically different practice contexts, this reflective practice proved crucial.

## Return to Uganda: context and preparation

When I returned to Hoima in February 2025, I found a well-planned opportunity. Important preparations had already been made, as Hoima Regional Referral Hospital had been chosen as the location for dialysis expansion:

- **Infrastructure:** A brand-new, cutting-edge intensive care unit with capacity for up to ten dialysis machines was built.
- **Workforce preparation**: Five nurses and clinical officers from Hoima completed a three-month dialysis-training program at Kiruddu National Referral Hospital in 2023. The same team participated in a 1-month refresher training upon my return to strengthen their abilities and implement current protocols.
- **Equipment pipeline**: Kiruddu National Referral Hospital has agreed to provide the initial dialysis equipment as part of its expansion plans, and additional machines are expected to be acquired through Ministry of Health procurement.

I therefore went back to an environment where the fundamental components – infrastructure, skilled staff, and government commitment – were already in place rather than beginning from scratch. It was my responsibility to offer clinical leadership, ensure quality and safety, and incorporate the new service into the hospital’s larger clinical and instructional missions.

## Implementation and outcomes

On 12 August 2025, Hoima Regional Referral Hospital’s dialysis unit was formally put into service. The implementation phase, which took place between February 2025 and February 2026, saw the direct application and adaptation of skills learned throughout the fellowship.

Table 1 demonstrates the growth of dialysis services. The unit grew from zero functional machines to five, with cumulative sessions reaching approximately 2340 by February 2026. The unit currently runs at least three hemodialysis sessions per day, 6 days per week, across five machines (≈ 90 sessions per week).

**Table 1:** Dialysis service growth.

Period	Functional machines	Estimated sessions/week	Notes
Pre-August 2025	0	0	No dialysis services available
August 2025 (unit commissioning)	3	~54	Initial machines donated from Kiruddu National Referral Hospital
February 2026	5	~90	Additional machines supplied through Ministry of Health procurement

Total sessions = 2340.

By February 2026, the dialysis unit was running at least three hemodialysis sessions per day across five machines, 6 days per week, providing an estimated 90-dialysis sessions weekly.

Table 2 provides further details on the dialysis unit expansion, showing the change from the pre-fellowship period (February 2025) to the current status (February 2026), including the addition of 5 functional machines, 90 sessions per week, 16 chronic HD patients maintained, and 7 AKI patients managed.

**Table 2:** Dialysis unit expansion.

Metric	Pre-fellowship (Feb 2025)	Current (Feb 2026)	Change
Functional dialysis machines	0	5	+ 5
Hemodialysis sessions/week	0	90	+ 90
Cumulative sessions (Aug 2025–Feb 2026)	0	~2340	+~2340
Chronic HD patients maintained	0	16	+16
AKI patients managed	0	7	+7

The unit currently runs at least three hemodialysis sessions per day, 6 days per week across five machines (≈90 sessions per week).

Table 3 summarizes patient outcomes. Among 7 AKI patients, 4 recovered, 2 died, and 1 case is ongoing. Among 20 CKD patients, 16 remain active, 2 died, and 2 were lost to follow-up. Two transplant referrals were made (one to Uganda, one to India).

**Table 3:** Patient outcomes.

Category	Number	Outcome
AKI	7	4 recovered, 2 died, 1 ongoing
CKD	20	16 active, 2 died, 2 lost to follow up
Transplant referrals	2	Uganda 1, India 1

### Educational impact

As part of a teaching hospital, Hoima’s new dialysis unit has become an integral training site:

- Nursing students: Forty-five diploma and degree nursing students have completed clinical rotations in the dialysis unit.
- Medical students: 28 medical students from Kampala International University have rotated through the unit.
- Continuing education: Weekly case discussions and monthly journal clubs have been established, drawing participants from medicine, nursing, and allied health professions.

This educational multiplier effect ensures that the impact of a single fellowship extends beyond direct patient care to shape the next generation of health workers.

## Discussion

The current paper shows how, when combined with institutional preparedness and national health policy support, international fellowship training may be converted into sustained clinical service development in a low-resource setting^[^11^]^. In this experience, dialysis services were established and expanded at a regional referral hospital in Uganda using nephrology fellowship training from a high-income nation, resulting in quantifiable improvements in access to renal replacement therapy, health worker training, and referral pathway development.

Circular capacity building, a model of global health training in which knowledge gained overseas is brought back to the home institution, adapted to local realities, and redistributed through teaching, service delivery, and system strengthening, is a crucial idea that emerged from this experience. Circular capacity building stresses the ongoing transfer of skills between contexts, which leads to the progressive strengthening of the local health system, in contrast to traditional training methods, where knowledge transfer may stay unidirectional^[^12,13^]^.

Overseas training by itself does not ensure better healthcare delivery in many LMICs, since returning specialists may face obstacles such as inadequate personnel, equipment shortages, or a lack of institutional support^[^14^]^. Training programs are most successful when they are connected to the infrastructure already in place and when local leadership is involved in their execution, according to earlier research in global health^[^15,16^]^. In the current environment, fellowship skills were quickly translated into clinical practice thanks to the availability of trained nurses, the provision of dialysis machines made possible by the national dialysis expansion program, and administrative dedication.

Tables 1 and 2 serve as examples of how service capacity can expand when equipment and qualified workers are made accessible simultaneously. The increase from zero dialysis sessions before 2025 to more than 2340 sessions in a single year is indicative of organizational improvement, including scheduling systems, nursing protocols, and supply management, as well as technical capabilities. This illustrates how capacity-building incorporates both clinical expertise and the structure of the health system.

Table 3 demonstrates the clinical impact of local dialysis availability. Patients who would not have otherwise had access to chronic dialysis were initiated on chronic dialysis, and patients with AKI who previously would have needed to be sent to distant centers could now receive care locally. Although mortality and loss to follow-up remain challenges, these outcomes are consistent with reports from other low-resource dialysis programs where late presentation and financial limitations affect survival^[^1,6,9^]^.

Table 4 represents another important component of circular capacity building. The training of nursing students, medical students, and junior doctors ensures that knowledge gained through the fellowship is not limited to a single individual but is shared within the institution. Educational meetings, audits, and case discussions help create a culture of continuous learning, which is essential for sustainability in settings with limited numbers of specialists.

**Table 4:** Training activities – opportunities to mentor and teach others.

Activity	Number
Nursing students	45
Medical students	28
Weekly meetings	Yes
Monthly audits	Yes

Table 5 (Adapting the Canadian Model to the Local Context on Return) illustrates how key components of the Canadian nephrology care model were pragmatically modified for the Ugandan setting. The Ugandan approach exhibits context-sensitive innovation: monthly renal team meetings replaced interdisciplinary rounds, standard patient education materials were reduced to pictorial guides for low-literacy patients, a dedicated nurse champion coordinated vascular access, and monthly audits targeted infection rates and dialysis adequacy. This reflects a shift from high-resource standardization to high-impact, resource-conscious implementation, preserving core principles while ensuring feasibility and sustainability in a low-resource setting.

**Table 5:** Adapting the Canadian model to the local context on return.

Canadian model	Hoima adaptation
Interdisciplinary rounds	Monthly “renal team meetings” with nephrology, nursing, pharmacy, and nutrition representatives
Patient education materials	Simplified pictorial guides for low-literacy patients;
Vascular access coordination	Dedicated nurse champion for access monitoring and referral coordination
Quality improvement framework	Monthly audit of dialysis adequacy and catheter-related bloodstream infections

This table illustrates how, after receiving specialized training in Canada, important components of the Canadian nephrology treatment model were pragmatically modified for the Ugandan setting. The Ugandan approach exhibits context-sensitive innovation, whereas Canada stresses organized, resource-rich multidisciplinary systems. Monthly renal team meetings were converted from interdisciplinary rounds, ensuring cooperative decision-making in spite of personnel constraints. To accommodate poor reading levels, standard patient education materials were reduced to graphical instructions. A committed nurse champion successfully localized vascular access coordination, which is normally system-driven in Canada, to expedite monitoring and referrals. In a similar vein, targeted monthly audits aimed at infection rates and dialysis adequacy were created using formal quality improvement frameworks.

Overall, this reflects a shift from high-resource standardization to high-impact, resource conscious implementation, preserving core principles while ensuring feasibility and sustainability in a low-resource setting.

Table 6 (Comparison of Other Forms of Training to the Circular Capacity Model) contrasts this model with other widely used global health training methodologies. Traditional foreign training programs may enhance individual skills but may not result in system transformation if the climate is not conducive to implementation. Although exchange programs offer exposure, they might not have a long-term local influence. In contrast, circular capacity building is possible even in settings with limited resources, since it depends on a mix of institutional preparedness, individual training, and adaptation to local conditions^[^17,18^]^.

**Table 6:** Comparison of other forms of training to circular capacity model.

Domain	Circular capacity building	Conventional models
Core concept	Bidirectional exchange with return and local implementation	Primarily unidirectional training or service delivery
Training flow	Train abroad → return home → adapt and implement locally	Train locally or via visiting faculty; limited external immersion
Knowledge transfer	Contextualized, adaptive to local systems	Often standardized curricula with variable local adaptation
Sustainability	High – builds durable local systems and leadership	Moderate – depends on continued external support or funding
Local ownership	Strong – led by locally trained specialists	Variable – sometimes externally driven or supported
Capacity building focus	Systems strengthening + service delivery + training	Clinical training and service delivery
Human resource development	Creates local champions and future trainers	Expands workforce but fewer system-level leaders
Resource utilization	Emphasizes innovation within constraints	May rely on external resources, faculty, or infrastructure
Continuity of care	Strong – returning specialists ensure continuity	Can be fragmented if reliant on short-term placements
Scalability	High if local leadership is sustained	Limited by funding, partnerships, and external personnel
Examples of outcomes	New programs, protocols, audits, training pipelines	Improved service delivery, increased workforce numbers

Table 7 (Adaptation of the Canadian Model to the Ugandan Context) demonstrates the process of adjusting procedures learned during fellowship to the local setting. For instance, established referral pathways took the place of formal transplant clinics, multidisciplinary rounds were transformed into monthly team meetings, and complex quality-improvement systems were reduced to routine audits. These modifications show that successful capacity building requires careful adjustment to fit existing resources rather than exact duplication of high-income approaches.

**Table 7:** Adaptation of Canadian model to the Ugandan context.

Canada	Hoima
Weekly multidisciplinary rounds	Monthly meetings
Dialysis education	Simplified guides
Access team	Nurse monitoring
QI programs	Monthly audit
Transplant clinic	Referral pathway

For specialties like nephrology, where treatment necessitates both specialist knowledge and technical support, the concept of cyclical capacity building may be especially pertinent. The increasing prevalence of kidney disease in Africa and other low-resource locations has prompted international studies to highlight the need for locally led solutions^[^2,14^]^. A workable approach to improving care without relying solely on outside missions may be provided through training programs that enable professionals to return to operational systems where they may put their talents to use^[^11^]^.

### Limitations

This report has several limitations. It depicts a relatively brief follow-up period (12 months) and represents the experience of a single-center. Rather than using a formal research methodology, outcomes were reported descriptively. The reported results are service measures rather than patient-level clinical outcomes (such as long-term survival or quality of life). Long-term viability after the initial launch phase remains unproven. Additionally, having a dedicated nephrologist at a regional hospital is still uncommon in Uganda; individual fellowships are insufficient to address workforce shortages of this magnitude. These findings may not be generalizable because it is a single-institution, single-fellow experience. The recorded difficulties – a 28.6% mortality rate in AKI patients and the death or follow-up attrition of four CKD patients (20% of the entire cohort) – are equally significant. These depressing numbers highlight the necessity of ongoing quality improvement, better infection control, and improved patient support systems and represent the realities of providing complex care in settings with limited resources^[^19^]^.

### Implications for global health training

These findings imply that training institutes in HICs that host foreign fellows should:

- Explicitly discuss adaptation and contextualization during training.
- Encourage fellows to create implementation plans before they return.
- Provide alumni networks and continued mentoring to support post-return success.
- Develop collaborations with domestic institutions rather than with individuals.
- For governments and institutions in LMICs:
- Strategic selection of trainees aligned with national health priorities is essential.
- Preparing the receiving environment before training optimizes the return on investment.
- Protected time and resources for returning fellows enable implementation.
- Integrating returning fellows into teaching positions multiplies impact.

## Conclusion

International fellowships may contribute to sustainable growth in the health system through circular capacity building^[^10^]^. When presented as reciprocal interactions and aligned with systemic preparedness, international medical fellowships have the potential to be potent catalysts for health system improvement. Within a year of the fellow’s return, a Canadian nephrology fellowship was translated into quantifiable gains at a regional hospital in Uganda, illustrating the potential of “circular capacity building.” The fellow’s ability to adapt, contextualize, lead change, and train others is just as important to success as clinical expertise. This methodology is broadly applicable to medical and surgical education in a variety of global health disciplines.

## Data Availability

Data supporting the findings of this study are available from the corresponding author (Louis Nyende, Lnyende@gmail.com) upon reasonable request.

## Research registration unique identifying number (UIN)

Researchregistry11898.
